# P-76. Risk factors associated with Gram-negative early hip and knee prosthetic joint infections: a single-center retrospective cohort study

**DOI:** 10.1093/ofid/ofae631.283

**Published:** 2025-01-29

**Authors:** Grace Cullen, Yuan Gu, Jorge Salinas, Daisuke Furukawa

**Affiliations:** Stanford University, Palo Alto, California; Stanford University, Palo Alto, California; University of Iowa Hospitals and Clinics, Stanford, California; Stanford University, Palo Alto, California

## Abstract

**Background:**

Prosthetic joint infection (PJI) is a devastating complication of total joint arthroplasty, occurring in 1-2% of all prosthetic joint surgeries. While rare, Gram-negative (GN) bacteria are important causes of PJIs and can be difficult to treat due to increasing antimicrobial resistance. We looked at cases of early PJIs due to GN bacteria in our institution to identify potential risk factors.

**Methods:**

We conducted a retrospective cohort study using the Center for Disease Control’s National Healthcare Safety Network data report of surgical site infections (SSI) for our institution between January 1, 2011 and June 1, 2023. Procedure codes HPRO and KPRO were used to identify total hip (THA) and knee (TKA) arthroplasty cases. Welch two sample t-test and Chi-square tests were used for between-group comparisons of continuous and categorical outcomes, respectively. Multiple logistic regression was used to identify risk factors and estimate adjusted odds ratios (aOR) for GN PJIs.

**Results:**

Our cohort included 17,725 arthroplasty cases, of which 174 developed a SSI of the implanted joint ≤90 days of index surgery, with a cumulative incidence of 0.98%. Of the SSI cases, 34 (20%) isolated GN bacteria while 140 (80%) were caused by other organisms. The most common GN bacteria were *E. coli* (11), *Klebsiella* species (8), *P. aeruginosa* (8). Patients with GN infections were older, with a median age of 70 (IQR 61-76) compared to 65 (IQR 58-70) in the non-GN infections group (p = 0.090). Duration of index surgery was longer in GN infections, with a median duration of 204 minutes (IQR 121-304) compared to 151 minutes (IQR 110-219, p=0.019). There were no differences between GN bacterial infections and infections due to other organisms for sex, American Society of Anesthesiologists physical status score, surgical risk score, body mass index, or history of diabetes. In the logistic regression, age and duration were associated with increased odds of GN infection with an aOR of 1.04 for age (p=0.05) and 1.05 for duration (p=0.003).

**Conclusion:**

The cumulative incidence of SSI of prosthetic hip and knee arthroplasties was low at < 1% with GN infections making up a minority of cases. Age at time of index surgery and duration of surgery were significantly associated with an increased risk of GN bacterial infection.

**Disclosures:**

**All Authors**: No reported disclosures

